# Applying artificial neural network for early detection of sepsis with intentionally preserved highly missing real-world data for simulating clinical situation

**DOI:** 10.1186/s12911-021-01653-0

**Published:** 2021-10-22

**Authors:** Yao-Yi Kuo, Shu-Tien Huang, Hung-Wen Chiu

**Affiliations:** 1 School of Medicine, College of Medicine, Taipei Medical University, Taipei, Taiwan; 2 Department of Emergency Medicine, Mackay Memorial Hospital, Taipei, Taiwan; 3 Graduate Institute of Biomedical Informatics, College of Medical Science and Technology, Taipei Medical University, Taipei, Taiwan

**Keywords:** Sepsis, Prediction, Artificial neural network, Machine learning, Artificial intelligence

## Abstract

**Purpose:**

Some predictive systems using machine learning models have been developed to predict sepsis; however, they were mostly built with a low percent of missing values, which does not correspond with the actual clinical situation. In this study, we developed a machine learning model with a high rate of missing and erroneous data to enable prediction under missing, noisy, and erroneous inputs, as in the actual clinical situation.

**Materials and methods:**

The proposed artificial neural network model was implemented using the MATLAB ANN toolbox, based on stochastic gradient descent. The dataset was collected over the past decade with approval from the appropriate institutional review boards, and the sepsis status was identified and labeled using Sepsis-3 clinical criteria. The imputation method was built by last observation carried forward and mean value, aimed to simulate clinical situation.

**Results:**

The mean area under the receiver operating characteristic (ROC) curve (AUC) of classifying sepsis and nonsepsis patients was 0.82 and 0.786 at 0 h and 40 h prior to onset, respectively. The highest model performance was found for one-hourly data, demonstrating that our ANN model can perform adequately with limited hourly data provided.

**Conclusions:**

Our model has the moderate ability to predict sepsis up to 40 h in advance under simulated clinical situation with real-world data.

## Introduction

Sepsis is a clinical syndrome caused by a dysregulated host response to infection [1]. This inflammatory response can lead to multiple organ dysfunction syndrome, including acute respiratory distress syndrome, acute renal failure, disseminated intravascular coagulation, and even death. For decades, sepsis has been considered challenging to treat in hospitals globally given its high mortality and high medical costs. Older patients aged ≥ 65 years account for the majority (60–85%) of all cases of sepsis, as older people are more susceptible to infection and have a higher risk of sepsis [2–5]. With the older population increasing worldwide, the incidence of sepsis may continue to increase, resulting in sepsis being a persistent, challenging problem. Identifying early sepsis, an early form of infection, is important to prevent sepsis progressing to severe condition such as severe sepsis or septic shock; each hour of delayed treatment is associated with an approximately 3.6–9.9% increase in mortality [6]. Furthermore, in areas of the world with the lowest socio-demographic index, the need for greater prevention of sepsis is highlighted by Global Burden of Disease Study 2017 [7], which emphasized the need to identify and to predict sepsis. However, no formal definition exists for early sepsis. Conflicting results have been provided for the ability of warning scores systems such as Quick Sepsis-Related Organ Failure Assessment (qSOFA) and National Early Warning Score (NEWS) to predict early sepsis [8–10]; patients may be misclassified as having sepsis based on their inflammation status, leading to a higher rate of antibiotic use and *Clostridioides difficile* infection, and antibiotic use did not affect 30-day mortality [11]. Moreover, in patients with systemic inflammatory response syndrome (SIRS) without evidence of infection, sepsis could not be predicted or identified, as SIRS is not always caused by infection [12].

Rapid progress has been made in machine learning in the last few years. Machine learning involves computer programs that undergo a learning process, with different rules attempted and learning performance improved. Machine learning is an influential and powerful tool for turning information into knowledge and is good at learning the rules governing a phenomenon [13]. Some studies have applied machine learning for data mining for diagnosing appendicitis [14] and diabetes [15] and for tumor assessment [16].

Applying machine learning with diverse variables and indicators has also been investigated. Akram’ s team used continuous (minute-by-minute) physiologic data to predict sepsis and demonstrated that salient physiomarkers are temporally and differentially expressed in septic patients [17]. Joseph’ team used physiologic, laboratory data and subjective variables to predict onset of vasopressor therapy and found that practice-specific features denoting measurement recency improved local performance [18]. An-Kwok’s team discussed the use of an expansive number of physiologic, laboratory, and demographic variables to create efficient, automated prediction of acute respiratory failure and acute respiratory distress syndrome [19].

Some predictive systems using machine learning models have been developed to predict or identify sepsis [20]. Gradient tree boosting models with 0% missing inputs using only vital signs can achieve the performance of 0.90 area under receiver operating characteristic (ROC) curve (AUC) when identifying sepsis and can achieve the performance of 0.84 AUC when predicting sepsis 24 h prior to onset [21, 22]. Logistic regression models using laboratory data with 7% missing inputs can achieve the performance of 0.83 AUC when identifying sepsis [23]. These models achieved favorable performance in the presence of low-percent missing and erroneous data. Nonetheless, in the actual clinical situation, missing and erroneous data exist due to several reasons. Some studies have reported that these missing and erroneous data have become a challenge for machine learning models to convert information into knowledge. The AUC of a gradient tree boosting model can decrease from 0.90 to 0.75 in the presence of 60% missing data [21].

Artificial neural network (ANN), a machine learning model, has been successfully used to solve highly difficult and complex problems in the field of physical sciences and in organizational research. ANN enables faster and efficient data collection and processing [24, 25]. Furthermore, as it is regarded as a practical and flexible modeling tool, ANN can generalize pattern information to new data, and it has information processing characteristics to learning power, high parallelism, fault tolerance, nonlinearity, noise tolerance, and capabilities of generalization [25]. One study used ANN to classify bacteremia and nonbacteremia patients with 20 clinical variables, including demograpahic variables, vital signs, and laboratory data. The AUC of prediction performance was 0.729 (95% confidence interval [CI]: 0.712–0.728) [26]. Another study used ANN for neonatal sepsis diagnosis with 25 maternal and neonatal features. The prediction performance was 0.933 in sensitivity, 0.800 in specificity and 0.944 in AUC [27].

Above published models could discriminate between sepsis and nonsepsis patients. However, a reliable model should be established to predict the sepsis onset timing in advance using before-sepsis-onset data with a high missing rate, corresponding to the actual clinical situation. Therefore, in this study, we developed a model based on ANN for sepsis prediction by using patient vital signs and laboratory data comprising up to 80% missing and erroneous data as the input, to see an easy shallow network is suitable to address these problems or not. First, we used ANN to classify sepsis and nonsepsis patients with different sepsis onset timings prior to onset. Second, we assessed how the different timings prior to onset affect prediction performance. Finally, we attempted to precisely predict the timing of sepsis onset.

## Materials and methods

### Datasets

The data used in this study were obtained from a public domain database, which consisted of Intensive Care Unit (ICU) patient records in Beth Israel Deaconess Medical Center and Emory University Hospital, including a total of 40,336 patient records, collected over the past decade with approval from the appropriate Institutional Review Boards [28]. Each record consisted of a combination of hourly vital sign summaries, laboratory values, and demographic variables. Specifically, the data contained 40 clinical variables: 8 vital sign variables, 26 laboratory variables, and 6 demographic variables. Tables 1 and 2 present these variables. We changed the definition of SepsisLabel to correspond to our experiment. A summary of vital signs and laboratory value data in the dataset is shown in Table 3. The missing rate of vital signs ranged from 9.88% (for the heart rate) to 66.16% (for temperature). Moreover, the missing rate of laboratory data ranged from 82.89% (for glucose) to 99.81% (for direct bilirubin). More details of the dataset are provided in a previous study [29].

**Table 1:** Clinical time series data: vital signs (rows 1–7), demographics (rows 8–13), and outcome (row 14)

	Measurement	Description
1	HR	Heart rate (beats per minute)
2	O_2_Sat	Pulse oximetry (%)
3	Temp	Temperature (°C)
4	SBP	Systolic BP (mm Hg)
5	MAP	Mean arterial pressure (mm Hg)
6	DBP	Diastolic BP (mm Hg)
7	Resp	Respiration rate (breaths per minute)
8	Age	Age (years)
9	Gender	Female (0) or male (1)
10	Unit1	Administrative identifier for ICU unit (MICU); false (0) or true (1)
11	Unit2	Administrative identifier for ICU unit (SICU); false (0) or true (1)
12	HospAdmTime	Time between hospital and ICU admission (hours since ICU admission)
13	ICULOS	ICU length of stay (hours since ICU admission)
14	SepsisLabel^*^	For septic patients, SepsisLabel is 1 if t ≥ t_sepsis_ and 0 if t < t_sepsis_
		For non-septic patients, SepsisLabel is 0

*We changed the definition of SepsisLabel to correspond to our experiment. The original definition is as follows: For septic patients, SepsisLabel is 1 if t ≥ t_sepsis_ − 6 and 0 if t < t_sepsis_ − 6. For nonsepsis patients, SepsisLabel is 0

**Table 2:** Clinical time series data: laboratory values

	Measurement	Description
1	EtCO_2_	End tidal carbon dioxide (mm Hg)
2	BaseExcess	Excess bicarbonate (mmol/L)
3	HCO_3_	Bicarbonate (mmol/L)
4	FiO_2_	Fraction of inspired oxygen (%)
5	pH	pH
6	PaCO_2_	Partial pressure of carbon dioxide from arterial blood (mm Hg)
7	SaO_2_	Oxygen saturation from arterial blood (%)
8	AST	Aspartate transaminase (IU/L)
9	BUN	Blood urea nitrogen (mg/dL)
10	Alkalinephos	Alkaline phosphatase (IU/L)
11	Calcium	Calcium (mg/dL)
12	Chloride	Chloride (mmol/L)
13	Creatinine	Creatinine (mg/dL)
14	Bilirubin direct	Direct bilirubin (mg/dL)
15	Glucose	Serum glucose (mg/dL)
16	Lactate	Lactic acid (mg/dL)
17	Magnesium	Magnesium (mmol/dL)
18	Phosphate	Phosphate (mg/dL)
19	Potassium	Potassiam (mmol/L)
20	Bilirubin total	Total bilirubin (mg/dL)
21	TroponinI	Troponin I (ng/mL)
22	Hct	Hematocrit (%)
23	Hgb	Hemoglobin (g/dL)
24	PTT	Partial thromboplastin time (s)
25	WBC	Leukocyte count (count/L)
26	Fibrinogen	Fibrinogen concentration (mg/dL)
27	Platelets	Platelet count (count/mL)

**Table 3:** Summary of vital signs and laboratory data in the datasets

Number of patients	40,336
Number of septic patients	2932
Sepsis prevalence	7.26%
Number of rows	1,424,147
Number of entries	10,486,913
Density of entries	19.9%

### Definition of sepsis onset time

We labeled patient data in accordance with the clinical criteria of Third International Consensus Definitions for Sepsis and Septic Shock. For each sepsis patient, we specified the following three time points to define the onset time t_sepsis_ of sepsis:

- t_suspicion_: Clinical suspicion of infection identified as the earlier timestamp of intravenous (IV) antibiotics and blood cultures within a given time interval. If IV antibiotics were given first, then the cultures must have been obtained within 24 h. If cultures were obtained first, then IV antibiotics must have been ordered within 72 h. In either case, IV antibiotics must have been administered for at least 72 consecutive hours.
- t_SOFA_: Occurrence of organ failure as identified by a 2-point increase in the Sequential Organ Failure Assessment (SOFA) score within a 24-h period.
- t_sepsis_: Onset of sepsis identified as being earlier than t_suspicion_ and t_SOFA_ as long as t_SOFA_ occurred no more than 24 h before or 12 h after t_suspicion_

### Data preprocessing

Missing values in the original data were intentionally preserved for conforming to the actual clinic situation. The missing values do become a challenge. Jang-Sikchoi’s team used logistic regression as the algorithm, and last observation carried forward and K-nearest neighbors as imputation methods for sepsis screening [23]. Ujjwol’ s team used gradient boosting tree as the algorithm and mean value as an imputation method for sepsis prediction [30]. To address this problem and to simulate the clinical situation, we imputed the missing values first by last observation carried forward. If the initial hourly data were unavailable for a variable in the patient record, the missing value was imputed with mean value calculated from the data for the variable in all 40,336 patient records. In addition to the original 40 variables, three new variables were created as follows: heart rate/systolic blood pressure, blood urea nitrogen/creatinine, and oxygen saturation from arterial blood/fraction of inspired oxygen.

### Machine learning model

ANN was used as our machine learning model in this study. ANN pattern recognition was implemented using the MATLAB ANN toolbox, which was based on stochastic gradient descent (SGD). SGD is an iterative method for optimizing an objective function with suitable smoothness properties (e.g., differentiable or subdifferentiable). It can be regarded as a stochastic approximation of gradient descent optimization, because it replaces the actual gradient (calculated from the entire dataset) with an estimate thereof (calculated from a randomly selected subset of the data). Particularly, in high-dimensional optimization problems, this reduces the computational burden, achieving faster iterations traded-off against a lower convergence rate.

We constructed a two-layer feed-forward network, with sigmoid hidden and softmax output neurons. The output layer was a softmax layer as an activation function outputting the probability of sepsis. The error function was evaluated based on cross-entropy and the percentage of misclassification errors.

**Fig. 1:**
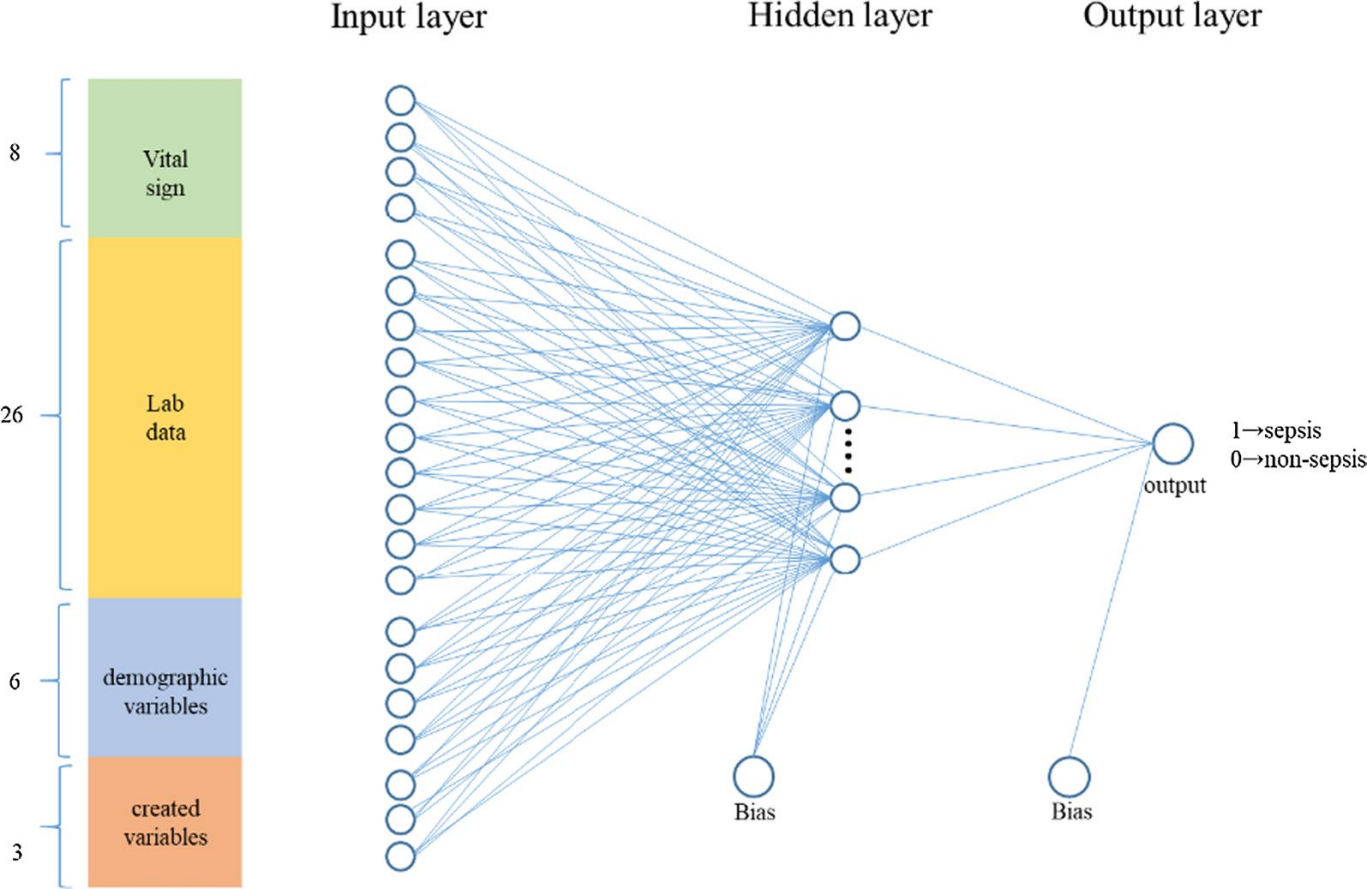
Schematic of the ANN model

The input of the classifier included all 43 variables: 8 vital sign variables, 26 laboratory variables, 6 demographic variables, and 3 created variables. To train our classifier, the number of hidden neurons was set as 100, 150 and 200 for one-hourly data; 300, 400 and 500 for three-hourly data; and 600, 700 and 800 for five-hourly data. The optimized number of hidden neurons was found by trial-and-error. Figure 1 provides the schematic of our ANN model.

### Experiment

#### Classifying sepsis and nonsepsis patients for predicting sepsis

In this study, 40,336 patients consisted of 2932 sepsis patients and 37,404 nonsepsis patients. When training artificial intelligence-based models with imbalanced data with significantly higher negative results than positive results, outcomes tend to be negative [31]. To address this problem, we adjusted the ratio of sepsis to nonsepsis patients to 1:1 by random matching. Next, to predict whether patients will develop sepsis, we extracted one-hourly data, three-hourly data, and five-hourly data prior to onset from all 2932 sepsis patients. How many hours prior to onset we would set depended on different experimental conditions. For example, in the one-hourly data experiment, the data of 1 h prior to sepsis onset were labeled as nonsepsis data initially in the dataset. We then defined the data to be sepsis data and used them to train our model to predict the status of sepsis 1 h in advance. We also randomly extracted one-hourly data, three-hourly data, and five-hourly data from 2932 nonsepsis patients, who were randomly matched to sepsis patients. These data were defined as nonsepsis data and used to predict the status of nonsepsis.

**Fig. 2:**
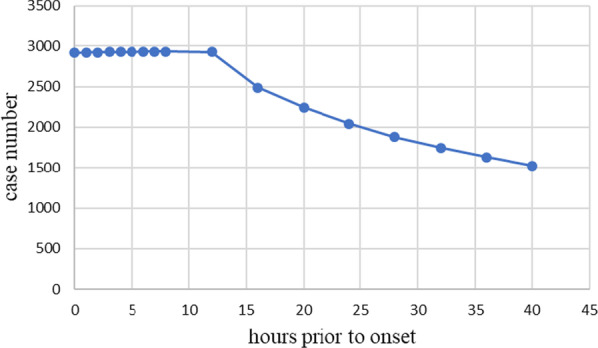
Case number of the one-hourly data experiment

In the one-hourly data experiment, the number of hidden neurons was set as 200. We extracted one-hourly data over 0–40 h prior to onset separately from sepsis patient records. The details of case numbers are provided in Fig. 2. Each set of one-hourly data consisted of 43 variables. Thus, the number of inputs was 43.

In the three-hourly data experiment, the number of hidden neurons was set as 500. We extracted three-hourly data over 1–3 to 13–15 h prior to onset separately from sepsis patient records. The 1–3-h three-hourly sepsis data consisted of the data of 1 h, 2 h and 3 h prior to onset. Each set of three-hourly data consisted of 129 variables (3 × 43). Thus, the number of inputs was 129.

In the five-hourly data experiment, the number of hidden neurons was set as 800. We extracted five-hourly data over 1–5 to 16–20 h prior to onset separately from sepsis patient records. The 1–5-h five-hourly sepsis data consisted of the data of 1 h, 2 h, 3 h, 4 h and 5 h prior to onset. Each set of five-hourly data consisted of 215 variables (5 × 43). Thus, the number of inputs was 215.

**Fig. 3:**
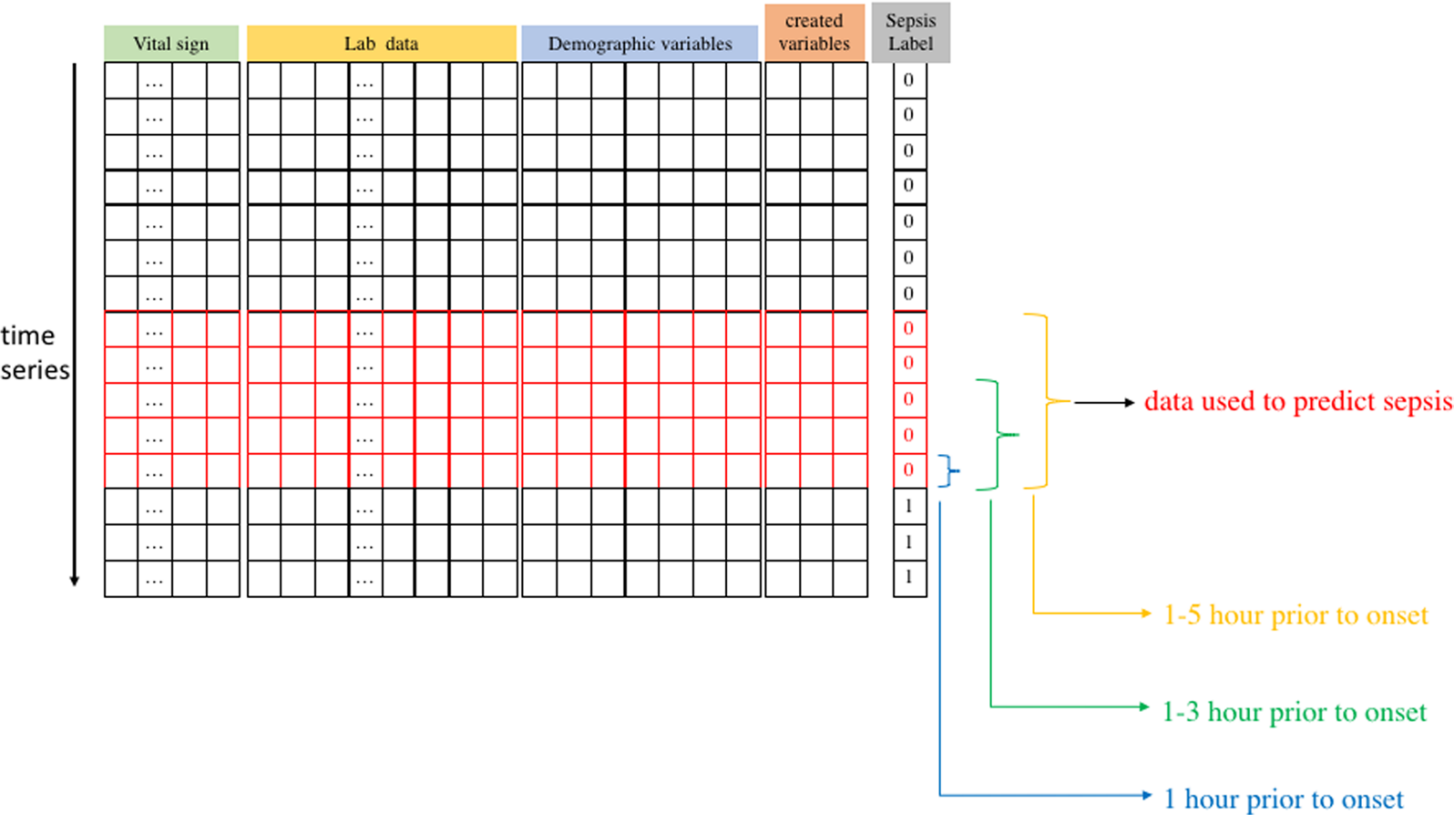
Schematic of the one-hourly data, three-hourly data, and five-hourly data experiments

Figure 3 shows the schematic of one-hourly, three-hourly, and five-hourly data experiments.

#### Using sepsis patient records only for predicting onset timing

**Fig. 4:**
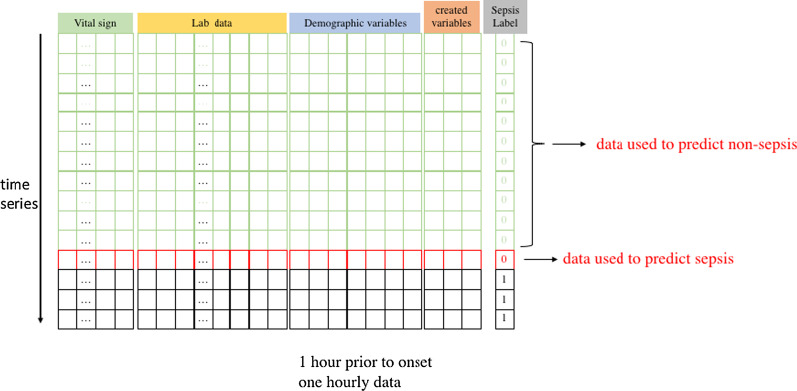
Schematic of the experiment using only sepsis patient records

We applied one-hourly data in this experiment, the number of hidden neurons was set as 200, and the number of inputs was 43. In sepsis patients, one-hourly data over 0–40 h prior to onset were extracted separately according to the experiment design; these data were termed as sepsis data. One-hourly data obtained prior to sepsis data were defined as nonsepsis data. We then adjusted the ratio of sepsis data to nonsepsis data to 1:1 by random matching. Figure 4 shows schematic of the experiment using only sepsis patient records.

### Model validation and performance measurement

We divided the dataset into two groups: 85% of the data into a training group and 15% into a testing group, in order to build the 85% training and 15% testing cross-validation method. The training group was presented to the network during training, and the network was adjusted according to its error. Furthermore, 17.6% of the training group was used in algorithm of Levenberg–Marquardt to prevent over-fitting. The testing group had no effect on training and provided an independent measure of network performance after training. The training process ended when the gradient of performance was less than 10^−6^. Finally, we chose an adequate and well-trained model according to its training performance and testing performance.

The model’s performance was determined using the area under the ROC curve (AUC) metric, sensitivity, and specificity. Sepsis and nonsepsis were set as positive and negative outcomes, respectively. We conducted all the experiments at a significance level of 95%.

## Results

### Classifying sepsis and nonsepsis patients for predicting sepsis

In each experiment, we trained 10 models with different random relative weights.

**Fig. 5:**
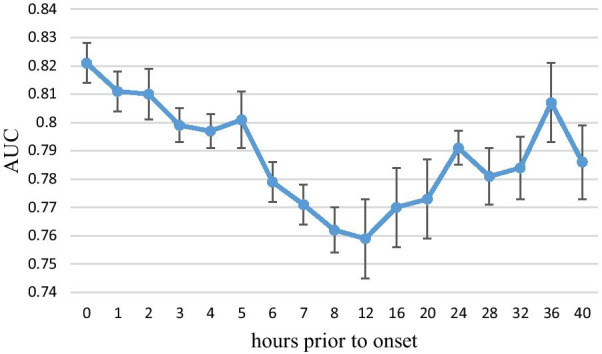
Performance characteristics of the one-hourly data experiment

**Fig. 6:**
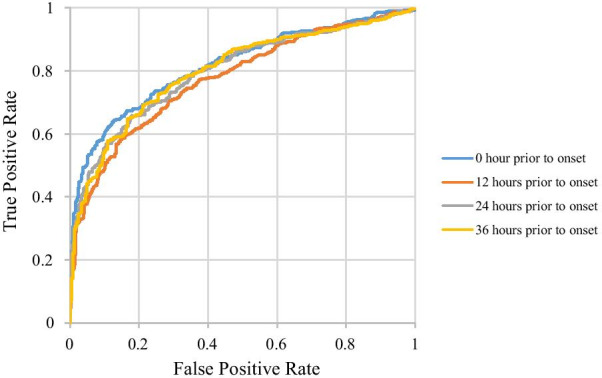
ROC curves of the one-hourly data experiment

Performance in the one-hourly data experiment is shown in Table 4, Figs. 5 and 6. The AUC of the training group was the highest at 0 h prior to onset, which was used for identifying sepsis, and the mean AUC was 0.82. With an increase in the number of hours prior to onset, performance started to decline, reaching the lowest mean AUC 0.76 at 12 h prior to onset. No significant difference was found in the AUC performance of 0, 1 and 2 h prior to onset. Furthermore, the performance of more than 12 h prior to onset started to increase. A rebounding effect of the AUC performance was observed between 13 and 40 h prior to onset. The mean AUC at 40 h prior to onset was 0.786. Thus, our ANN model has the moderate ability to predict whether patients will develop sepsis, even up to 40 h prior to sepsis onset. This ability can enable clinical health professionals to take appropriate measures beforehand to treat sepsis.

**Table 4:** Performance characteristics of the one-hourly data experiment

Hours prior to onset	AUC (95% CI)	Accuracy	Sensitivity	Specificity
0	0.821 (0.814–0.828)	0.758	0.836	0.678
6	0.779 (0.772–0.787)	0.731	0.793	0.669
12	0.759 (0.745–0.773)	0.703	0.772	0.633
24	0.791 (0.785–0.797)	0.738	0.817	0.657
36	0.807 (0.793–0.822)	0.742	0.793	0.690

**Fig. 7:**
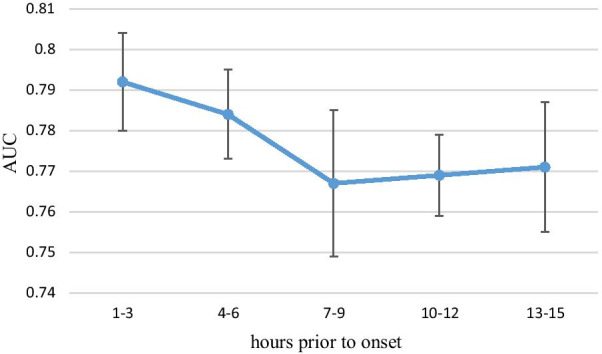
Performance characteristics of the three-hourly data experiment

Performance in the three-hourly data experiment is shown in Table 5 and Fig. 7. The AUC of the training group was the highest at 1–3 h prior to onset, with a mean AUC of 0.792. Compared with the one-hourly data experiment, a higher performance was not found for the three-hourly data experiment. Although the AUC performance was the lowest (with mean AUC of 0.767) at 7–9 h prior to onset and a rebounding effect of the AUC performance was observed between 7–9 and 13–15 h prior to onset, no significant difference was found for the AUC performance of all three-hourly data experiments.

**Table 5:** Performance characteristics of the three-hourly data experiment

Hours prior to onset	AUC (95% CI)	Accuracy	Sensitivity	Specificity
1–3	0.792 (0.780–0.805)	0.730	0.796	0.665
4–6	0.784 (0.773–0.794)	0.721	0.777	0.665
7–9	0.767 (0.749–0.784)	0.703	0.754	0.654
10–12	0.769 (0.759–0.779)	0.711	0.762	0.660
13–15	0.771 (0.755–0.787)	0.716	0.775	0.642

**Fig. 8:**
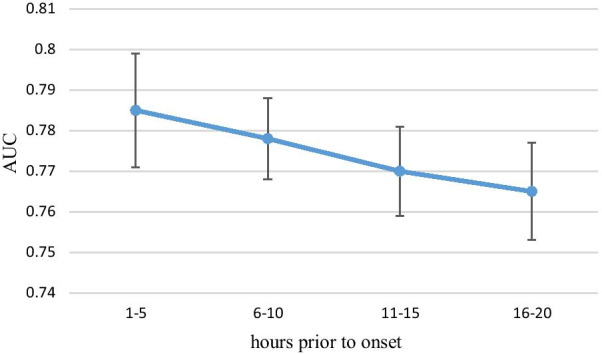
Performance characteristics of the five-hourly data experiment

Performance in the five-hourly data experiment is presented in Table 6 and Fig. 8. The AUC of the training group was the highest at 1–5 h prior to onset, with a mean AUC of 0.785. Compared with the one-hourly data experiment and three-hourly data experiment, a higher performance was not found for the five-hourly data experiment. The rebounding effect of AUC was not observed. With an increase in the number of hours prior to onset, performance kept declining, reaching the lowest mean AUC of 0.765 at 16–20 h prior to onset. No significant difference was observed in the AUC performance of all five-hourly data experiments.

**Table 6:** Performance characteristics of the five-hourly data experiment

Hours prior to onset	AUC (95% CI)	Accuracy	Sensitivity	Specificity
1–5	0.785 (0.771–0.800)	0.719	0.762	0.676
6–10	0.778 (0.768–0.788)	0.716	0.764	0.668
11–15	0.770 (0.759–0.781)	0.713	0.775	0.652
16–20	0.765 (0.753–0.778)	0.713	0.758	0.668

Overall, the highest performance was found for one-hourly data experiment for identifying and predicting sepsis, demonstrating that our ANN model can perform adequately with limited hourly data provided.

### Using sepsis patient records only for predicting onset timing

**Fig. 9:**
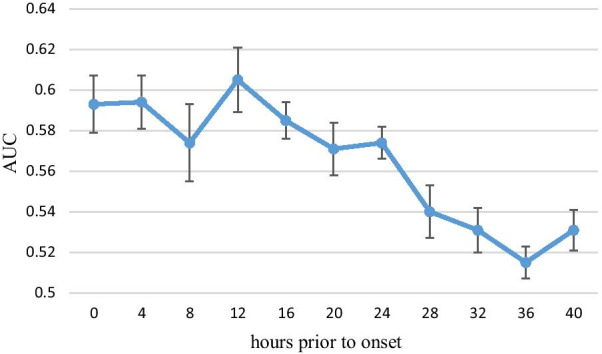
Performance characteristics of the experiment using only sepsis patient records

The performance of the experiment using only sepsis patient records is shown in Table 7 and Fig. 9. The mean AUC of the testing group ranged between 0.605 and 0.515. Compared with the experiment for classifying sepsis and nonsepsis patients, the performance of the experiment using only sepsis patient records was much lower, demonstrating that our ANN model is not suitable for precisely predicting the onset timing of sepsis or classifying the status of the same patient at different time point. Furthermore, no significant difference was found in the results at 0–24 h prior to onset, nor were any differences found in the results at 28–40 h prior to onset. However, the results at 0–24 h prior to onset significantly outperformed the results at 28–40 h prior to onset, demonstrating that the data closer to sepsis onset had more predictive value.

**Table 7:** Performance characteristics of the experiment using only sepsis patient records

Hours prior to onset	AUC (95% CI)	Accuracy	Sensitivity	Specificity
0	0.593 (0.579–0.607)	0.572	0.572	0.548
4	0.594 (0.581–0.607)	0.573	0.590	0.556
8	0.574 (0.555–0.594)	0.563	0.587	0.539
12	0.605 (0.589–0.621)	0.586	0.611	0.561
24	0.574 (0.566–0.582)	0.548	0.557	0.540
36	0.515 (0.503–0.527)	0.523	0.517	0.529

## Discussion

### Classifying sepsis and nonsepsis patients for predicting sepsis

In clinical situations, it is unlikely that the complete data of every hour would be available. Missing and erroneous data are unavoidable under normal circumstances. Nonetheless, our ANN model showed a performance above 0.8 AUC in the presence of up to 80% missing and erroneous data, showing its ability of clinical application and proving that the model can predict whether patients will develop sepsis before sepsis onset and before significant changes in vital signs and laboratory data.

In our experiment, we could even predict whether patients would develop sepsis up to 40 h in advance prior to sepsis onset, with a performance of 0.786 AUC. Although this ANN model cannot precisely predict sepsis onset, it can identify patients who will develop sepsis 40 h in advance, which is valuable information for clinical and medical professionals. Therefore, they can provide adequate management and treatment 40 h in advance, including early source control, fluid therapy, vasoactive medications, and antibiotic administration [32]. According to some autopsy studies in adults, the most common error in the treatment of sepsis is the delay in diagnosing sepsis and infection treatment; this delay is avoidable if we are aware of the sepsis status of the patient in advance [33, 34]. The Surviving Sepsis Campaign (SCC), a joint collaboration between the European Society of Intensive Care Medicine, International Sepsis Forum, and the Society of Critical Care Medicine, has also emphasized the importance of early source control and antibiotic administration [35]. Furthermore, SCC has shown that compliance with adequate early resuscitation and management bundle could significantly reduce sepsis mortality in hospitals [36]. Many studies have shown the benefits and advantages of early medical intervention for sepsis, with the early identification of sepsis. Therefore, our ANN model can be applied in clinical settings to provide sepsis onset prediction for clinical and medical professionals.

In the one-hourly data, three-hourly data, and five-hourly data experiments, we found adequate performance of the one-hourly data for classifying sepsis and nonsepsis patients in advance. The AUC performance of the one-hourly data experiment from 1 to 6 h prior to onset was between 0.797 and 0.811. The AUC performance of the three-hourly data experiment from 1 to 6 h prior to onset was between 0.784 and 0.792. The AUC performance of the five-hourly data experiment from 1 to 5 h prior to onset was 0.785. More hourly data as the input did not increase the performance of the model. Therefore, our ANN model only needs the initial one-hourly data, demonstrating that we can assess the sepsis risk of a patient with the initial vital signs and laboratory data.

Other studies have used machine learning models to classify sepsis patients and nonsepsis patients. Qingqing’s team used gradient tree boosting as the algorithm and vital signs as the input. They observed a mean AUC of 0.90 at 0 h prior to onset at a 0% missing rate and a mean AUC of 0.75 at 0 h prior to onset at a 60% missing rate [21]. Christopher’ team used gradient tree boosting as the algorithm and vital signs as the input. At a 0% missing rate, they observed a mean AUC of 0.88 at 0 h prior to onset, mean AUC of 0.84 at 24 h prior to onset, and mean AUC of 0.83 at 48 h prior to onset. However, the case number was only 375 and 147 at 24 and 48 h prior to onset, respectively [22]. Jang-Sikchoi’s team used logistic regression as the algorithm and laboratory data as the input. They observed a mean AUC of 0.83 at 0 h prior to onset at a 7% missing rate [23]. Compared with the models in these studies, our ANN model provides more advantages in clinical situations, as our model was trained with data with an 80% missing rate and imputed under clinical situation.

### Using sepsis patient records only for predicting onset timing

In this experiment, we used our ANN model to classify every hourly dataset of sepsis patients. We aimed to find out whether any significant differences exist in vital signs and laboratory data before sepsis onset, which we could use to precisely predict the timing of sepsis onset. However, favorable performance was not found in this experiment, with the highest mean AUC of 0.6. Even though we tried to classify the hourly data when sepsis occurred, the mean AUC reached only 0.593, demonstrating that our model is not suitable for classifying every hourly dataset of sepsis patient. Therefore, our ANN model is not suitable for precisely predicting the timing of sepsis onset. An algorithm consisting of time series might be considered to build a model to predict the precise timing of sepsis onset.

## Conclusions

In the experiment using sepsis patient and nonsepsis patient records, the mean AUC reached 0.821. Our ANN model has the moderate ability to predict whether patients will develop sepsis, even up to 40 h prior to sepsis onset under simulated clinical situation with real-world data. In addition, this might imply the presence of a significant difference between sepsis patients and nonsepsis patients, even at 40 h prior to sepsis onset. Nonetheless, in sepsis patients, regardless of how many hours prior to onset, a significant difference was not found in vital signs and laboratory data. This might have resulted in the poor performance of our ANN model.

The results showed the effectiveness of our ANN model for early classifying sepsis and nonsepsis patient. However, the predictive performance still needed to be improved. We hope to cope with this issue by optimizing the models, using novel imputation methods and pursuing new features closely related to sepsis such as monocyte distribution width [37]. In our ANN model, we have demonstrated that given one-hourly input data can identify and predict sepsis and the accuracy is comparable to given three-hourly and five-hourly input data, which need extra information from the patients, and the necessity of more hourly data as input will be further investigated in the future.

## Limitation

With an increase in the number of hours prior to onset, the case number would decrease because some patient records would not include the hourly data that were long time before the onset of sepsis. It was unclear whether this would affect our results. The patient records including hourly data long time before the onset of sepsis may have more similar patterns, creating difficulty in evaluating the predication performance of our ANN model.

To validate the mean and last observation carried forward method and perform the noise tolerance capability of our ANN model, an experiment using the dataset with no missing or erroneous values should be performed. However, the dataset consisting of laboratory data from blood test is difficult to have no missing value in clinical situation. Therefore, there is no hourly data consisting of no missing values for all variables before imputation in our dataset, and we cannot perform this experiment.

In comparison to other machine learning models, the “black box” nature of ANNs acts as a barrier in providing biological interpretation of the model. We can hardly present the value that the variables provide, relation between variables and results, and the threshold of making a decision. Furthermore, ANN needs more data for training, for it consists of many hidden neurons, which means that more parameters are needed to figure out.

## Data Availability

All patient records files are available from the PhysioNet Computing in Cardiology Challenge 2019 (https://doi.org/10.13026/v64v-d857).
